# 2-Amino-4-nitro­phenol monohydrate

**DOI:** 10.1107/S1600536810035415

**Published:** 2010-09-11

**Authors:** Hasan Tanak, Ferda Erşahin, Metin Yavuz, Orhan Büyükgüngör

**Affiliations:** a Department of Physics, Faculty of Arts & Science, Ondokuz Mayıs University, TR-55139 Kurupelit-Samsun, Turkey; b Department of Chemistry, Faculty of Arts & Science, Ondokuz Mayıs University, 55139 Samsun, Turkey

## Abstract

The title compound, C_6_H_6_N_2_O_3_·H_2_O, crystallizes with two formula units in the asymmetric unit. The mol­ecules are essentially planar with the nitro groups twisted slightly out of the ring planes [maximum deviations from the ring plane of 0.13 (2) and 0.22 (2) Å in the two mol­ecules]. The respective O—N—C—C torsion angles are 6.0 (4) and 12.5 (4)°. In the crystal structure, mol­ecules are linked by inter­molecular N—H⋯O, C—H⋯O, O—H⋯O and O—H⋯N inter­actions into a three-dimensional network.

## Related literature

For the use of nitro­aromatics as inter­mediates in explosives, dyestuffs, pesticides and organic synthesis, see: Yan *et al.* (2006 ▶). For the occurrence of nitro­aromatics in industrial wastes and as direct pollutants in the environment, see: Yan *et al.* (2006 ▶); Soojhawon *et al.* (2005 ▶). For related structures, see: Tanak *et al.* (2010 ▶); Bi *et al.* (2009 ▶); Garden *et al.* (2004 ▶). 
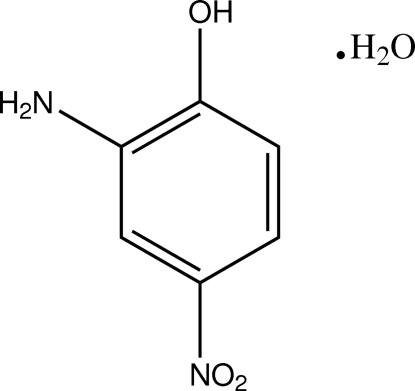


## Experimental

### 

#### Crystal data

C_6_H_6_N_2_O_3_·H_2_O
*M* *_r_* = 172.14
Monoclinic, 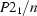

*a* = 7.539 (5) Å
*b* = 21.436 (5) Å
*c* = 9.714 (5) Å
β = 99.328 (5)°
*V* = 1549.1 (13) Å^3^
*Z* = 8
Mo *K*α radiation
μ = 0.13 mm^−1^
*T* = 296 K
0.62 × 0.30 × 0.05 mm

#### Data collection

Stoe IPDS II diffractometer
Absorption correction: integration (*X-RED32*; Stoe & Cie, 2002 ▶) *T*_min_ = 0.578, *T*_max_ = 0.892
8719 measured reflections
3031 independent reflections
1598 reflections with *I* > 2σ(*I*)
*R*_int_ = 0.069

#### Refinement

*R*[*F* ^2^ > 2σ(*F* ^2^)] = 0.054
*wR*(*F* ^2^) = 0.116
*S* = 0.98
3031 reflections
242 parameters
H atoms treated by a mixture of independent and constrained refinement
Δρ_max_ = 0.17 e Å^−3^
Δρ_min_ = −0.21 e Å^−3^

### 

Data collection: *X-AREA* (Stoe & Cie, 2002 ▶); cell refinement: *X-AREA*; data reduction: *X-RED32* (Stoe & Cie, 2002 ▶); program(s) used to solve structure: *SHELXS97* (Sheldrick, 2008 ▶); program(s) used to refine structure: *SHELXL97* (Sheldrick, 2008 ▶); molecular graphics: *ORTEP-3 for Windows* (Farrugia, 1997 ▶); software used to prepare material for publication: *WinGX* (Farrugia, 1999 ▶).

## Supplementary Material

Crystal structure: contains datablocks I, global. DOI: 10.1107/S1600536810035415/bt5342sup1.cif

Structure factors: contains datablocks I. DOI: 10.1107/S1600536810035415/bt5342Isup2.hkl

Additional supplementary materials:  crystallographic information; 3D view; checkCIF report

## Figures and Tables

**Table 1:** Hydrogen-bond geometry (Å, °)

*D*—H⋯*A*	*D*—H	H⋯*A*	*D*⋯*A*	*D*—H⋯*A*
N2—H4⋯O2^i^	0.89 (5)	2.51 (5)	3.382 (3)	165 (4)
N2—H5⋯O3^ii^	0.89 (5)	2.47 (5)	3.317 (3)	159 (4)
N4—H10⋯O4^iii^	0.92 (5)	2.18 (5)	3.062 (3)	159 (4)
N4—H11⋯O6^iv^	0.88 (5)	2.28 (5)	3.064 (3)	147 (4)
O7—H13⋯N2^ii^	0.89 (5)	2.00 (5)	2.877 (4)	172 (5)
O7—H14⋯O8^v^	0.78 (5)	2.43 (5)	3.166 (4)	158 (5)
O8—H15⋯O2^vi^	0.80 (5)	2.55 (5)	3.102 (3)	127 (4)
O8—H15⋯O5^vi^	0.80 (5)	2.35 (5)	3.038 (3)	144 (5)
O8—H16⋯N4^iv^	0.96 (5)	1.88 (5)	2.821 (4)	164 (4)
C6—H6⋯O1^i^	0.93	2.47	3.304 (4)	150
C12—H12⋯O4^iii^	0.93	2.54	3.254 (4)	133
O3—H1⋯O8	0.82	1.85	2.657 (3)	169
O6—H7⋯O7	0.82	1.81	2.619 (4)	168

Symmetry codes: (i) 
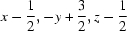
; (ii) 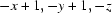; (iii) 
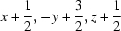
; (iv) 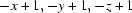; (v) 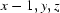; (vi) 
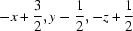
.

**Fig. 1.**
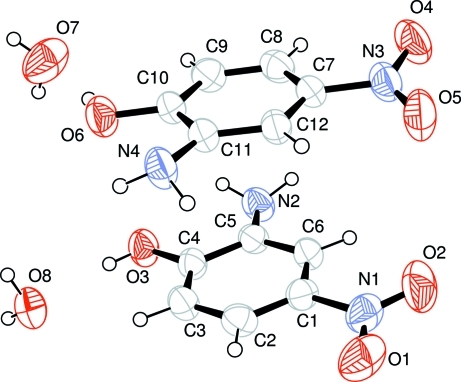
The molecular structure of the title compound, showing the atom-numbering scheme and 50% probability diplacement ellipsoids.

**Fig. 2.**
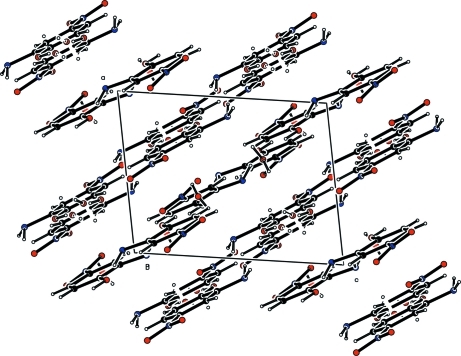
The crystal packing of the title compound.

## supplementary crystallographic information

### Comment

Nitroaromatics are widely used either as materials or as intermediates in explosives, dyestuffs, pesticides and organic synthesis (Yan *et al.*, 2006). Nitroaromatics occur as industrial wastes and direct pollutants in the environment, and are relatively soluble in water and detectable in rivers, ponds and soil (Yan *et al.*, 2006; Soojhawon *et al.*, 2005).

There is two independent molecules in the asymmetric unit of the title compound (I, Fig. 1). The bond lengths and angles in (I) have normal values, and are comparable with those in the related structures (Tanak *et al.*, 2010; Bi *et al.*, 2009; Garden *et al.*, 2004). The aromatic ring systems are almost planar with the maximum deviation, 0.13 (2) Å for atom O1 in the ring system C1—C6 and -0.22 (2) Å for atom O4 in the ring system C7—C12.

In the crystal structure, the molecules are linked by intermolecular N—H···O, C—H···O, O—H···O and O—H···N interactions (see Table I) into a three-dimensional network.

### Experimental

The commercially available compound (Acros organics) was recrystallized from ethanol.

### Refinement

C-bound H atoms were positioned geometrically and refined using a riding model, with C—H = 0.93 Å and *U*_iso_(H) = 1.2*U*_eq_(C). The position of the H4, H5, H10, H11, H13, H14, H15 and H16 atoms were obtained from a difference map and these atoms were freely refined. The H atoms of the hydroxyl groups were refined using a riding model with O-H = 0.82Å and *U*_iso_(H) = 1.5*U*_eq_(O).

### Crystal data

**Table:**

C_6_H_6_N_2_O_3_·H_2_O	*F*(000) = 720
*M**_r_* = 172.14	*D*_x_ = 1.476 Mg m^−^^3^
Monoclinic, *P*2_1_/*n*	Mo *K*α radiation, λ = 0.71069 Å
Hall symbol: -P 2yn	Cell parameters from 7016 reflections
*a* = 7.539 (5) Å	θ = 1.9–27.3°
*b* = 21.436 (5) Å	µ = 0.13 mm^−^^1^
*c* = 9.714 (5) Å	*T* = 296 K
β = 99.328 (5)°	Prism, yellow
*V* = 1549.1 (13) Å^3^	0.62 × 0.30 × 0.05 mm
*Z* = 8	

### Data collection

**Table:**

Stoe IPDS II diffractometer	3031 independent reflections
Radiation source: fine-focus sealed tube	1598 reflections with *I* > 2σ(*I*)
graphite	*R*_int_ = 0.069
Detector resolution: 6.67 pixels mm^-1^	θ_max_ = 26.0°, θ_min_ = 1.9°
rotation method scans	*h* = −7→9
Absorption correction: integration (*X-RED32*; Stoe & Cie, 2002)	*k* = −26→26
*T*_min_ = 0.578, *T*_max_ = 0.892	*l* = −11→11
8719 measured reflections	

### Refinement

**Table:**

Refinement on *F*^2^	Secondary atom site location: difference Fourier map
Least-squares matrix: full	Hydrogen site location: inferred from neighbouring sites
*R*[*F*^2^ > 2σ(*F*^2^)] = 0.054	H atoms treated by a mixture of independent and constrained refinement
*wR*(*F*^2^) = 0.116	*w* = 1/[σ^2^(*F*_o_^2^) + (0.0426*P*)^2^] where *P* = (*F*_o_^2^ + 2*F*_c_^2^)/3
*S* = 0.98	(Δ/σ)_max_ < 0.001
3031 reflections	Δρ_max_ = 0.17 e Å^−^^3^
242 parameters	Δρ_min_ = −0.21 e Å^−^^3^
0 restraints	Extinction correction: *SHELXL97* (Sheldrick, 2008), Fc^*^=kFc[1+0.001xFc^2^λ^3^/sin(2θ)]^-1/4^
Primary atom site location: structure-invariant direct methods	Extinction coefficient: 0.0063 (11)

### Special details

**Table:**

Geometry. All e.s.d.'s (except the e.s.d. in the dihedral angle between two l.s. planes) are estimated using the full covariance matrix. The cell e.s.d.'s are taken into account individually in the estimation of e.s.d.'s in distances, angles and torsion angles; correlations between e.s.d.'s in cell parameters are only used when they are defined by crystal symmetry. An approximate (isotropic) treatment of cell e.s.d.'s is used for estimating e.s.d.'s involving l.s. planes.
Refinement. Refinement of *F*^2^ against ALL reflections. The weighted *R*-factor *wR* and goodness of fit *S* are based on *F*^2^, conventional *R*-factors *R* are based on *F*, with *F* set to zero for negative *F*^2^. The threshold expression of *F*^2^ > σ(*F*^2^) is used only for calculating *R*-factors(gt) *etc*. and is not relevant to the choice of reflections for refinement. *R*-factors based on *F*^2^ are statistically about twice as large as those based on *F*, and *R*- factors based on ALL data will be even larger.

### Fractional atomic coordinates and isotropic or equivalent isotropic displacement parameters (Å^2^)

**Table:**

	*x*	*y*	*z*	*U*_iso_*/*U*_eq_	
O3	0.6819 (3)	0.50238 (8)	0.1354 (2)	0.0700 (6)	
H1	0.7078	0.4727	0.1882	0.105*	
C11	0.4215 (3)	0.62410 (11)	0.4251 (3)	0.0524 (7)	
O6	0.3203 (3)	0.52487 (8)	0.3513 (2)	0.0738 (6)	
H7	0.2634	0.5061	0.2849	0.111*	
C4	0.7466 (3)	0.55594 (11)	0.2003 (3)	0.0525 (7)	
C5	0.7094 (3)	0.61040 (11)	0.1216 (3)	0.0521 (6)	
C12	0.4337 (3)	0.68701 (11)	0.4013 (3)	0.0534 (7)	
H12	0.4950	0.7129	0.4699	0.064*	
O8	0.7879 (3)	0.39992 (9)	0.2795 (3)	0.0796 (7)	
C2	0.9053 (4)	0.61399 (12)	0.3917 (3)	0.0581 (7)	
H2	0.9702	0.6158	0.4816	0.070*	
C10	0.3296 (3)	0.58634 (11)	0.3190 (3)	0.0540 (7)	
N4	0.4880 (4)	0.59777 (11)	0.5561 (3)	0.0661 (7)	
N2	0.6190 (4)	0.60576 (11)	−0.0165 (3)	0.0656 (7)	
C1	0.8706 (4)	0.66709 (11)	0.3123 (3)	0.0543 (7)	
C6	0.7737 (4)	0.66635 (11)	0.1799 (3)	0.0567 (7)	
H6	0.7515	0.7033	0.1299	0.068*	
O1	1.0411 (4)	0.72657 (11)	0.4830 (3)	0.0981 (8)	
C3	0.8408 (4)	0.55795 (12)	0.3337 (3)	0.0580 (7)	
H3	0.8612	0.5214	0.3853	0.070*	
N3	0.3654 (4)	0.77812 (11)	0.2558 (3)	0.0690 (7)	
N1	0.9413 (4)	0.72598 (12)	0.3708 (3)	0.0744 (8)	
C9	0.2545 (4)	0.61152 (12)	0.1923 (3)	0.0612 (7)	
H9	0.1966	0.5858	0.1219	0.073*	
C8	0.2650 (4)	0.67473 (12)	0.1700 (3)	0.0614 (7)	
H8	0.2126	0.6922	0.0856	0.074*	
C7	0.3544 (4)	0.71151 (11)	0.2748 (3)	0.0540 (7)	
O5	0.4692 (4)	0.80874 (9)	0.3407 (3)	0.0955 (8)	
O4	0.2702 (3)	0.80249 (9)	0.1563 (3)	0.0911 (8)	
O2	0.9015 (4)	0.77391 (10)	0.3045 (3)	0.1136 (10)	
O7	0.1271 (4)	0.45374 (12)	0.1652 (4)	0.1135 (11)	
H11	0.513 (6)	0.558 (2)	0.552 (5)	0.170*	
H5	0.544 (7)	0.574 (2)	−0.025 (5)	0.170*	
H10	0.584 (7)	0.620 (2)	0.603 (5)	0.170*	
H4	0.564 (7)	0.641 (2)	−0.048 (5)	0.170*	
H15	0.808 (7)	0.368 (2)	0.243 (6)	0.170*	
H16	0.686 (7)	0.394 (2)	0.327 (5)	0.170*	
H13	0.201 (7)	0.432 (2)	0.122 (6)	0.170*	
H14	0.055 (7)	0.431 (2)	0.188 (6)	0.170*	

### Atomic displacement parameters (Å^2^)

**Table:**

	*U*^11^	*U*^22^	*U*^33^	*U*^12^	*U*^13^	*U*^23^
O3	0.0838 (15)	0.0491 (10)	0.0692 (13)	−0.0074 (9)	−0.0118 (11)	0.0001 (10)
C11	0.0552 (17)	0.0446 (14)	0.0573 (18)	0.0031 (11)	0.0091 (13)	0.0025 (13)
O6	0.0843 (16)	0.0441 (10)	0.0885 (15)	−0.0017 (9)	0.0006 (11)	−0.0001 (10)
C4	0.0559 (17)	0.0441 (14)	0.0555 (18)	−0.0012 (12)	0.0031 (13)	−0.0018 (14)
C5	0.0512 (16)	0.0507 (14)	0.0534 (17)	0.0033 (12)	0.0056 (13)	−0.0005 (14)
C12	0.0575 (18)	0.0436 (14)	0.0573 (18)	0.0006 (11)	0.0038 (13)	0.0024 (13)
O8	0.0979 (19)	0.0521 (11)	0.0887 (18)	0.0038 (11)	0.0145 (13)	0.0065 (11)
C2	0.0568 (17)	0.0612 (17)	0.0530 (17)	0.0036 (13)	−0.0011 (13)	−0.0075 (15)
C10	0.0549 (18)	0.0402 (13)	0.068 (2)	0.0042 (11)	0.0126 (14)	0.0023 (14)
N4	0.0814 (19)	0.0477 (12)	0.0641 (17)	0.0015 (12)	−0.0041 (13)	0.0101 (13)
N2	0.0727 (18)	0.0609 (14)	0.0581 (16)	0.0057 (12)	−0.0050 (13)	0.0039 (13)
C1	0.0514 (16)	0.0478 (14)	0.064 (2)	−0.0011 (12)	0.0099 (14)	−0.0135 (14)
C6	0.0570 (17)	0.0458 (14)	0.068 (2)	0.0048 (12)	0.0128 (15)	0.0029 (15)
O1	0.0942 (18)	0.0970 (17)	0.0998 (19)	−0.0290 (13)	0.0057 (15)	−0.0430 (16)
C3	0.0661 (19)	0.0492 (14)	0.0557 (19)	0.0042 (12)	0.0007 (14)	0.0026 (13)
N3	0.0799 (19)	0.0529 (14)	0.0747 (19)	0.0083 (13)	0.0136 (15)	0.0146 (15)
N1	0.0694 (19)	0.0599 (17)	0.096 (2)	−0.0080 (13)	0.0205 (17)	−0.0282 (17)
C9	0.0613 (18)	0.0606 (16)	0.0597 (18)	0.0016 (13)	0.0039 (14)	−0.0047 (15)
C8	0.0599 (19)	0.0641 (17)	0.0594 (19)	0.0103 (14)	0.0075 (14)	0.0096 (15)
C7	0.0526 (17)	0.0459 (13)	0.0635 (19)	0.0067 (12)	0.0097 (14)	0.0088 (14)
O5	0.122 (2)	0.0524 (12)	0.104 (2)	−0.0134 (12)	−0.0068 (16)	0.0133 (13)
O4	0.1057 (19)	0.0673 (13)	0.0949 (18)	0.0214 (12)	−0.0002 (14)	0.0300 (13)
O2	0.137 (2)	0.0485 (13)	0.149 (3)	−0.0033 (13)	0.0030 (19)	−0.0150 (15)
O7	0.105 (2)	0.0883 (18)	0.156 (3)	−0.0325 (14)	0.0474 (18)	−0.0539 (18)

### Geometric parameters (Å, °)

**Table:**

O3—C4	1.361 (3)	N4—H11	0.88 (5)
O3—H1	0.8200	N4—H10	0.92 (5)
C11—C12	1.374 (3)	N2—H5	0.89 (5)
C11—C10	1.402 (4)	N2—H4	0.89 (5)
C11—N4	1.408 (4)	C1—C6	1.372 (4)
O6—C10	1.359 (3)	C1—N1	1.450 (3)
O6—H7	0.8200	C6—H6	0.9300
C4—C3	1.374 (4)	O1—N1	1.219 (4)
C4—C5	1.398 (3)	C3—H3	0.9300
C5—C6	1.380 (3)	N3—O4	1.223 (3)
C5—N2	1.406 (4)	N3—O5	1.230 (3)
C12—C7	1.380 (4)	N3—C7	1.444 (3)
C12—H12	0.9300	N1—O2	1.224 (3)
O8—H15	0.80 (5)	C9—C8	1.377 (4)
O8—H16	0.96 (5)	C9—H9	0.9300
C2—C1	1.376 (4)	C8—C7	1.374 (4)
C2—C3	1.382 (4)	C8—H8	0.9300
C2—H2	0.9300	O7—H13	0.89 (5)
C10—C9	1.378 (4)	O7—H14	0.78 (5)
			
C4—O3—H1	109.5	H5—N2—H4	112 (4)
C12—C11—C10	118.8 (2)	C6—C1—C2	122.7 (2)
C12—C11—N4	121.5 (3)	C6—C1—N1	118.9 (3)
C10—C11—N4	119.6 (2)	C2—C1—N1	118.4 (3)
C10—O6—H7	109.5	C1—C6—C5	119.5 (2)
O3—C4—C3	123.7 (2)	C1—C6—H6	120.2
O3—C4—C5	115.2 (2)	C5—C6—H6	120.2
C3—C4—C5	121.1 (2)	C4—C3—C2	120.4 (3)
C6—C5—C4	118.4 (2)	C4—C3—H3	119.8
C6—C5—N2	122.5 (2)	C2—C3—H3	119.8
C4—C5—N2	119.0 (2)	O4—N3—O5	122.0 (2)
C11—C12—C7	119.5 (3)	O4—N3—C7	119.0 (3)
C11—C12—H12	120.3	O5—N3—C7	119.1 (3)
C7—C12—H12	120.3	O1—N1—O2	121.6 (3)
H15—O8—H16	109 (4)	O1—N1—C1	119.6 (3)
C1—C2—C3	117.9 (2)	O2—N1—C1	118.7 (3)
C1—C2—H2	121.1	C8—C9—C10	120.0 (3)
C3—C2—H2	121.1	C8—C9—H9	120.0
O6—C10—C9	123.9 (3)	C10—C9—H9	120.0
O6—C10—C11	115.3 (2)	C7—C8—C9	118.8 (3)
C9—C10—C11	120.9 (2)	C7—C8—H8	120.6
C11—N4—H11	113 (3)	C9—C8—H8	120.6
C11—N4—H10	112 (3)	C8—C7—C12	122.0 (2)
H11—N4—H10	112 (4)	C8—C7—N3	120.3 (3)
C5—N2—H5	110 (3)	C12—C7—N3	117.7 (3)
C5—N2—H4	113 (3)	H13—O7—H14	109 (5)
			
O3—C4—C5—C6	−178.9 (2)	C5—C4—C3—C2	−1.8 (4)
C3—C4—C5—C6	1.6 (4)	C1—C2—C3—C4	0.6 (4)
O3—C4—C5—N2	−3.1 (4)	C6—C1—N1—O1	−173.4 (3)
C3—C4—C5—N2	177.4 (3)	C2—C1—N1—O1	6.0 (4)
C10—C11—C12—C7	0.6 (4)	C6—C1—N1—O2	5.2 (4)
N4—C11—C12—C7	−175.0 (2)	C2—C1—N1—O2	−175.4 (3)
C12—C11—C10—O6	−178.8 (2)	O6—C10—C9—C8	177.8 (2)
N4—C11—C10—O6	−3.2 (4)	C11—C10—C9—C8	−1.7 (4)
C12—C11—C10—C9	0.8 (4)	C10—C9—C8—C7	1.3 (4)
N4—C11—C10—C9	176.4 (3)	C9—C8—C7—C12	0.0 (4)
C3—C2—C1—C6	0.8 (4)	C9—C8—C7—N3	−179.0 (2)
C3—C2—C1—N1	−178.6 (2)	C11—C12—C7—C8	−1.0 (4)
C2—C1—C6—C5	−0.9 (4)	C11—C12—C7—N3	178.0 (2)
N1—C1—C6—C5	178.4 (2)	O4—N3—C7—C8	12.1 (4)
C4—C5—C6—C1	−0.3 (4)	O5—N3—C7—C8	−168.4 (3)
N2—C5—C6—C1	−175.9 (3)	O4—N3—C7—C12	−166.9 (3)
O3—C4—C3—C2	178.7 (2)	O5—N3—C7—C12	12.5 (4)

### Hydrogen-bond geometry (Å, °)

**Table:**

*D*—H···*A*	*D*—H	H···*A*	*D*···*A*	*D*—H···*A*
N2—H4···O2^i^	0.89 (5)	2.51 (5)	3.382 (3)	165 (4)
N2—H5···O3^ii^	0.89 (5)	2.47 (5)	3.317 (3)	159 (4)
N4—H10···O4^iii^	0.92 (5)	2.18 (5)	3.062 (3)	159 (4)
N4—H11···O6^iv^	0.88 (5)	2.28 (5)	3.064 (3)	147 (4)
O7—H13···N2^ii^	0.89 (5)	2.00 (5)	2.877 (4)	172 (5)
O7—H14···O8^v^	0.78 (5)	2.43 (5)	3.166 (4)	158 (5)
O8—H15···O2^vi^	0.80 (5)	2.55 (5)	3.102 (3)	127 (4)
O8—H15···O5^vi^	0.80 (5)	2.35 (5)	3.038 (3)	144 (5)
O8—H16···N4^iv^	0.96 (5)	1.88 (5)	2.821 (4)	164 (4)
C6—H6···O1^i^	0.93	2.47	3.304 (4)	150
C12—H12···O4^iii^	0.93	2.54	3.254 (4)	133
O3—H1···O8	0.82	1.85	2.657 (3)	169
O6—H7···O7	0.82	1.81	2.619 (4)	168

Symmetry codes: (i) *x*−1/2, −*y*+3/2, *z*−1/2; (ii) −*x*+1, −*y*+1, −*z*; (iii) *x*+1/2, −*y*+3/2, *z*+1/2; (iv) −*x*+1, −*y*+1, −*z*+1; (v) *x*−1, *y*, *z*; (vi) −*x*+3/2, *y*−1/2, −*z*+1/2.
